# Plant growth promotion and biocontrol properties of a synthetic community in the control of apple disease

**DOI:** 10.1186/s12870-024-05253-8

**Published:** 2024-06-13

**Authors:** Rongye Qiao, Mingzhen Xu, Jihang Jiang, Zhen Song, Meibin Wang, Lei Yang, Hui Guo, Zhiquan Mao

**Affiliations:** 1 State Key Laboratory of Efficient Production of Forest Resources, Beijing Forestry University, Beijing, 100083 China; 2 Institute of Environment and Sustainable Development in Agriculture, Chinese Academy of Agricultural Sciences, Beijing, China; 3 College of Horticulture Science and Engineering, Shandong Agricultural University, Taian, China; 4 National Engineering Research Center of Tree Breeding and Ecological Restoration, Beijing, 100083 China

**Keywords:** Plant growth promoting rhizobacteria (PGPR), Beneficial microbes, Biological control, Synthetic community (SynCom), Apple disease

## Abstract

**Background:**

Apple Replant Disease (ARD) is common in major apple-growing regions worldwide, but the role of rhizosphere microbiota in conferring ARD resistance and promoting plant growth remains unclear.

**Results:**

In this study, a synthetic microbial community (SynCom) was developed to enhance apple plant growth and combat apple pathogens. Eight unique bacteria selected via microbial culture were used to construct the antagonistic synthetic community, which was then inoculated into apple seedlings in greenhouse experiments. Changes in the rhizomicroflora and the growth of aboveground plants were monitored. The eight strains, belonging to the genera *Bacillus* and *Streptomyces*, have the ability to antagonize pathogens such as *Fusarium oxysporum*, *Rhizoctonia solani*, *Botryosphaeria ribis*, and *Physalospora piricola*. Additionally, these eight strains can stably colonize in apple rhizosphere and some of them can produce siderophores, ACC deaminase, and IAA. Greenhouse experiments with *Malus hupehensis* Rehd indicated that SynCom promotes plant growth (5.23%) and increases the nutrient content of the soil, including soil organic matter (9.25%) and available K (1.99%), P (7.89%), and N (0.19%), and increases bacterial richness and the relative abundance of potentially beneficial bacteria. SynCom also increased the stability of the rhizosphere microbial community, the assembly of which was dominated by deterministic processes (|β NTI| > 2).

**Conclusions:**

Our results provide insights into the contribution of the microbiome to pathogen inhibition and host growth. The formulation and manipulation of similar SynComs may be a beneficial strategy for promoting plant growth and controlling soil-borne disease.

**Supplementary Information:**

The online version contains supplementary material available at 10.1186/s12870-024-05253-8.

## Background

Apple replant disease (ARD) is a major problem for the apple industry worldwide, including in the Bohai Gulf region of China, where a large number of apple trees are planted. Continuous replanting severely affects the yield and quality of apple trees and causes serious economic losses. The act of replanting is often associated with increased inoculum levels and elevated activity of soil-borne plant pathogens, as well as disturbances in soil microbial communities, leading to reduced yields in apple cultivars over time [1]. ARD, primarily affecting apple roots, is significantly influenced by the complex interactions within the rhizosphere, the soil region near plant roots. The aboveground performance of plants is closely correlated with changes in underground microbial communities. The rhizosphere microbial community differs depending on the planting time and planting cycle, which in turn affect the physical and chemical properties of the subsoil and the growth of aboveground plants [2]. The composition and diversity of microbial communities play a pivotal role in influencing soil structure and biological interactions [3]. Variations in the rhizosphere microbial community may lead to microecological imbalances in the root zone of apple trees, and these imbalances could potentially be one of the factors contributing to the incidence of apple diseases. In a previous study, we used high-throughput sequencing to analyze the microbial communities of the rhizospheres of perennial apple trees around Bohai Gulf. The results revealed that replanting led to an increase in populations of potential pathogenic fungi such as *Verticillium* and bacteria such as *Xanthomonadaceae*, alongside a decrease in potentially beneficial bacterial populations like *Pseudomonas* and *Bacillus* [4]. Some studies have also found that replanting coincided with a rise in antagonistic bacteria and fungi, e.g., *Arthrobacter*, *Chaetomium*, indicating that heightened pathogen levels may induce increased microbial antagonism [5].

Apple replant diseases can be controlled by physical, chemical, and biological means. Biocontrol includes bioprospecting for new, active isolates but also an understanding of the mechanisms of pathogen antagonism, to allow their improvement and broader use. *Fusarium oxysporum*, *Rhizoctonia solani*, *Botryosphaeria ribis*, and *Physalospora piricola* are associated with soil-borne and plant diseases. *Rhizoctonia solani* is a major fungal pathogen responsible for ARD, particularly noted for its impact on apple production. *Fusarium oxysporum* has been identified as a pathogen causing rown and root rot in apples [6, 7]. *Botryosphaeria ribis* is linked with apple stem canker and fruit rot [8, 9]. Meanwhile, *Physalospora piricola* is known to cause bull’s-eye rot in apples, affecting fruit quality and storability [10, 11]. All four are typically present in the soil and in residual roots or crowns, where they cause a reduction in plant productivity. Conversely, many strains of *Bacillus* and *Streptomyces* are considered biocontrol agents, because they effectively colonize the rhizosphere of different plants species, including fruit trees, and produce a wide range of antimicrobial agents that can survive harsh environments [12]. Different strains of *Streptomyces* and *Bacillus* have thus been investigated for their ability to control fungal and bacterial diseases of plants, such as bacterial leaf blight caused by *Xanthomonas* [13, 14]. However, most of the current biological control methods are based on individual strains that exhibit limited resistance. For instance, *Bacillus thuringiensis*, extensively utilized as a biopesticide in agriculture, targets lepidopteran pests, beetles, and flies [15]. The antagonistic effects of a combination of multiple bacterial strains still need to be explored.

A synthetic community (SynCom) is designed by mixing selected strains with the aim of increasing microbial community stability through synergistic interactions between members in a manner that benefits plants. This approach also enables a detailed assessment of host and microbe characteristics under controlled, reproducible conditions [16]. SynCom construction is an essential step in verifying microbiome function and in studying the interactions between the microbiome and host plant. The isolation and culture of microorganisms can link amplicon sequencing data to functional validation and are key to elucidating the interactions between microbiome and host plant [17]. However, little research has examined the use of SynCom in combating apple diseases. The effects of SynCom on the growth of *Malus hupehensis* Rehd and the beneficial and harmful microorganisms in its rhizosphere are not known.

In this study, the responses of a microbial community to the application of different exogenous microorganisms were investigated, including the antagonism between rhizosphere bacteria and pathogens, the role of SynCom in plant growth, and the effect of SynCom on the rhizosphere microbial community and vice versa. These topics were explored by screening bacteria capable of inhibiting typical apple disease pathogens, which resulted in the isolation and characterization of eight isolates that were further assessed for their potential as biological control agents. Then a SynCom constructed using these resistant bacteria was tested in colonization and plant growth promotion experiments, to identify possible beneficial effects on *Malus hupehensis* Rehd. The structure, species composition, co-occurrence network characteristics, and assembly process of the microbial community were investigated, together with the changes in the responses to different treatments, to analyze how SynCom functions from a microbial perspective. Our results contribute new insights into the prevention and control of ARD and other apple diseases, offering preliminary steps towards developing an ecologically friendly and sustainable apple industry.

## Materials and methods

### Rhizosphere soil sampling

The rhizosphere soil in this study was collected from perennial apple trees in apple orchards around Bohai Gulf (China) in five sampling sites (Qixia, Muping, Laizhou, Huludao and Changli). The roots were shaken vigorously to remove loose soil and the 1–2 mm thick soil layer surrounding the root was defined as rhizosphere soil. To collect the rhizosphere soil, the root samples were transferred to sterile 50 mL centrifuge tubes containing 20 mL sterile 10 mM PBS and placed in a full-temperature shaker at 120 rpm/min, where they were oscillated for 20 min at room temperature [18]. The root system in each tube was removed with sterile tweezers, and the remaining suspension was centrifuged at high speed (6,000 × g, 4 °C) for 20 min. All soil samples were stored at 4 °C.

### Bacterial isolation and assessment of antimicrobial and plant growth promotion (PGP) activity

Rhizosphere bacteria were isolated and antimicrobial activity of rhizosphere bacterial isolates was tested against the apple pathogens *Fusarium oxysporum*, *Rhizoctonia solani* (AG-5), *Botryosphaeria ribis*, and *Physalospora piricola*, and apple pathogens are provided by the microbiology laboratory of Shandong Agricultural University. To prepare a 10^6^-fold dilution of soil for microbial analysis, a 1 g soil sample is suspended in 99 mL of sterile water, mixed thoroughly to ensure homogeneity. This suspension is then serially diluted to achieve the desired dilution factor. From this suspension, 100 µL is spread onto Luria-Bertani (LB) agar plates and incubated overnight at 30 °C. Representative colonies differing in color, shape, and size are selected and subcultured onto fresh LB plates for an additional 2 days to obtain pure cultures [19, 20].

Strains of *Fusarium oxysporum*, *Rhizoctonia solani*, *Botryosphaeria ribis*, and *Physalospora piricola* were cultured on potato dextrose agar and incubated at 28 °C for 7 days. Then dual culture assays on PDA were conducted by placing a square agar disk (side length of 0.5 mm) containing mycelium of the pathogens at the center of the plate. Then, two 6 mm wells were created on opposite sides of the plate, and 100 µL of culture medium from each bacterial strain, after 2 days of incubation in LB liquid medium (10 g NaCl, 10 g tryptone, 5 g yeast dip powder, 1 L of distilled water, pH 7.0-7.5), was pipetted into the wells. The plates were incubated at 30 °C for 3 days and the antagonistic activity was estimated by measuring the growth inhibition zone [21]. Antagonistic bacteria with inhibitory effects on all four pathogens were selected for further study.

The antagonistic bacterial strains were then evaluated for inhibition between isolates. Different strains were streaked pairwise on LB agar plates without intersecting, and the growth process was observed for any antagonistic phenomena [22]. The selected bacterial strains were also evaluated for siderophore, indoleacetic acid (IAA), and ACC deaminase production. Siderophore-producing bacteria were screened by inoculating candidate strains onto chrome azurol S medium, incubating the plates at 30 °C in the dark for 2 days, and observing the size of the resulting orange halo [23]. ACC deaminase production was determined based on the ability of each candidate strain to use ACC (1-aminocyclopropane-1-carboxylate) as the sole nitrogen source. IAA and IAA-like molecules were quantitatively determined using a colorimetric Salkowski assay [24].

### Taxonomic identification and phylogenetic analysis of the strains

The TIANamp bacterial DNA kit (TIANGEN BIOTECH(BEIJING)CO., LTD) was used to extract the DNA of the antagonistic strains. Target fragments amplified using 16 S rDNA universal primers (27 F: 5′-CAGAGTTTGATCCTGGCT-3′, 1492R: 5′-AGGAGGTGATCCAGCCGCA-3′) served as the template for PCR amplification. The PCR reaction protocol begins with an initial denaturation step at 95 °C for 3 min to fully denature the DNA. This is followed by 35 cycles, each consisting of three steps: denaturation at 95 °C for 30 s, annealing at 52 °C for 30 s, and extension at 72 °C for 1 min. The process concludes with a final extension at 72 °C for 5 min to ensure that any remaining DNA is fully extended [25]. The amplified products were sequenced by Sangon Biotech (Shanghai) Co. Ltd., and the obtained sequences were used in a BLAST search (http://www.ncbi.nlm.nih.gov/) to identify the species of the isolates. The BLAST results were downloaded and the sequences were used to construct a phylogenetic tree using MEGA7.0 [26].

### Assembly and colonization of the SynCom

Eight bacterial strains with a highly antagonistic effect on *Fusarium oxysporum* (ZOI > 6 mm), *Rhizoctonia solaniI* (ZOI > 6 mm), *Botryosphaeria ribis* (ZOI > 8 mm), and *Physalospora piricola* (ZOI > 6 mm) were selected as candidate strains for SynCom construction. An equal volume of each strain (~ 10^8^ cells/mL) was mixed to establish the SynCom.

The persistence and viability of the isolates in the soil were tested using antibiotic resistance marking method. The labeled eight strains were inoculated in equal numbers in pots containing *Malus hupehensis* Rehd, and the rhizosphere-colonizing bacteria were recovered after inoculation. Each sample was diluted in sterile water and inoculated in medium supplemented with rifampicin and ampicillin. The results were assessed after 4 days of incubation at 30 °C and are reported as CFU/g rhizosphere soil [27].

### Pot experiment

The antagonistic ability of SynCom in the apple rhizosphere under natural conditions was also investigated in pot experiments using *Malus hupehensis* Rehd. *Malus huphensis* Rehd has deep roots and is commonly used as rootstock in major apple-producing regions of China. These seedlings are propagated through non-fusion techniques, avoiding crossbreeding, which ensures that the experimental material is genetically consistent. Antagonistic strains obtained from the screening were cultured in LB liquid medium at 28 °C and 170 rpm for 2 days, diluted with sterile water, and mixed in equal proportions to obtain a 10^8^ CFU·mL^− 1^ SynCom suspension [28, 29]. The pathogen suspension was composed of a combination of four fungi: *Fusarium oxysporum*, *Rhizoctonia solani*, *Botryosphaeria ribis*, and *Physalospora piricola*. Each of these fungi was individually inoculated into Potato Dextrose Broth (PDB) culture medium and incubated in a shaking incubator at 28 °C and 170 rpm for a period of 5–7 days. Following incubation, each fungal culture was filtered to obtain spore suspensions, which were then diluted with sterile water to achieve a concentration of 10^6^ spores/mL [30]. Subsequently, equal volumes of these diluted spore suspensions were combined to prepare the pathogen suspension. The root surfaces of healthy *Malus hupehensis* Rehd seedlings were washed and then immersed in the suspensions, depending on the treatment (see below) for 30 min. Then the seedlings were transferred to plastic pots (diameter of 29.6 cm) containing 2 kg mixed growing substrate consisting of soil from the apple orchard and sterilized nutrient soil in a 1:1 ratio. Watering management was carried out for 3 months according to conventional cultivation.

Four treatments were established in triplicate: inactivated SynCom suspension (25 mL) + sterile water (25 mL) (S treatment), pathogen suspension (25 mL) + sterile water (25 mL) (P), pathogen suspension (25 mL) + SynCom suspension (25 mL) (M), and SynCom suspension (25 mL) + sterile water (25 mL) (A). Root inoculation was started 3 months after seedling transplantation. The suspension was applied three times separated by a 1-week interval (The final concentration of bacteria added was about 3.75 × 10^7^ cells per gram of soil, and that of fungi was about 3.75 × 10^4^ spores per gram of soil) and the seedlings were harvested after 75 days. The biological characteristics (root length, plant height, the number of leaves) were determined 15, 45, and 75 days after application.

### Physicochemical measurements of pot soil

The treated *Malus hupehensis* Rehd seedlings were harvested after 75 days and potting soil was collected. Soil adhering to the roots, approximately 1 mm thick, was defined as rhizosphere soil. The soil pH, moisture content, organic matter content, and concentrations of available N, P, and K were measured [31].

### DNA extraction, amplification, and sequencing

Total DNA was extracted from 0.6–0.8 g soil using the FastDNA®SPIN kit (MP Biomedicals, Solon, USA) according to the manufacturer’s protocol. The primers 338F (5’-ACTCCTACGGGAGGCAGCA-3’) and 806R (5’-GGACTACHVGGGTWTCTAAT-3’) were used to amplify the V3–V4 region of bacterial 16 S rRNA, and the primers ITS1F (5’- CTTGGTCATTTAGAGGAAGTAA − 3’) and ITS1R (5’- GCTGCGTTCTTCATCGATGC − 3’) were used to amplify the ITS regions of fungi. Sequencing of purified PCR products was performed on an Illumina MiSeq PE300 platform at Major Biomedical Technology Co., Ltd (Shanghai, China). Subsequent to sequencing, raw data were assembled and quality-filtered following the methodology described by Caporaso [32], and chimeric sequences were removed using the QIIME2 tool. Sequences corresponding to mitochondria and chloroplasts were also removed. The remaining effective sequences were clustered into operational taxonomic units (OTUs) at 97% similarity [33]. The raw sequencing data were deposited in the Sequence Read Archive at NCBI with the accession number PRJNA1009678 and were under processed.

### Effects of SynCom on soil microbial community

The species diversity and richness of rhizosphere soil samples following SynCom application were characterized by calculating alpha-diversity indices for the microbial community. Microbial community composition was ordinated by principal coordinates analysis (PCoA) based on Bray-Curtis distances, and differences in rhizosphere soil samples after the application of SynCom and/or pathogens were compared using nonparametric permutational multivariate analysis of variance, based on the Adonis function in the R package [34]. Linear discriminant analysis and a significance test were used to explore the most discriminating genus between treatments, using linear discriminant analysis effect size (LefSe) [35]. A co-occurrence network was also constructed and the null-model was used to quantify community assembly processes. Environmental factors were combined with microbial communities to identify the biomarkers and environmental drivers in the different treatments. The predicted functional annotations were based on the PICRUSt and FUNGuild [36, 37].

### Statistical analyses

All statistical analyses were performed in the R environment. Student’s t tests (two-sided) were used to compare pairs of samples for significant differences. An analysis of variance (ANOVA) and Tukey’s honest significant difference (HSD) test were performed to determine significant differences in multiple comparisons, using the R agricolae package. Normality was assessed using the Shapiro-Wilk test, and homogeneity of variances was evaluated by Bartlett’s test.

Alpha-diversity indices, including the Richness, Shannon index, Chao1 index and Simpson index, were calculated using the “vegan” package in R. The results of PCoA based on a Bray–Curtis distance matrix were visualized using the “ggplot2” package, and the coordinates were used to draw 2D graphical outputs [38].

The R package “psych” was used to generate a species correlation matrix and to calculate correlations between OTUs based on Spearman analyses. The node and edge files were exported using Gephi, which was also used to further map the network. Network correlation parameters were also calculated using Gephi. Microbial community assembly was analyzed using the “NST0,” “picante,” and “ape” packages to calculate βNTI indices and the MEGA7.0 software to construct phylogenetic trees. The “Hmisc” and “picante” packages were used to calculate Bloomburg K values [39].

The physicochemical properties of soils driving microorganisms were analyzed using the “linkET” and “dplyr” packages. The “vegan,” “RandomForest,” and “reshape” packages were used to identify biomarkers and environmental drivers.

Function categorization based on KEGG (Kyoto Encyclopedia of Genes and Genomes) pathways were performed by PICRUSt according to a standard analysis process [36, 40–42]. The relative abundance of level 2 pathways was obtained, and the results were visualized using the RStudio. Fungal function was predicted using the FUNGuild tool of Majorbio Cloud [43].

## Results

### Assembly and characterization of simplified bacterial communities in rhizosphere soils of apple trees from the Bohai Gulf area

**Fig. 1:**
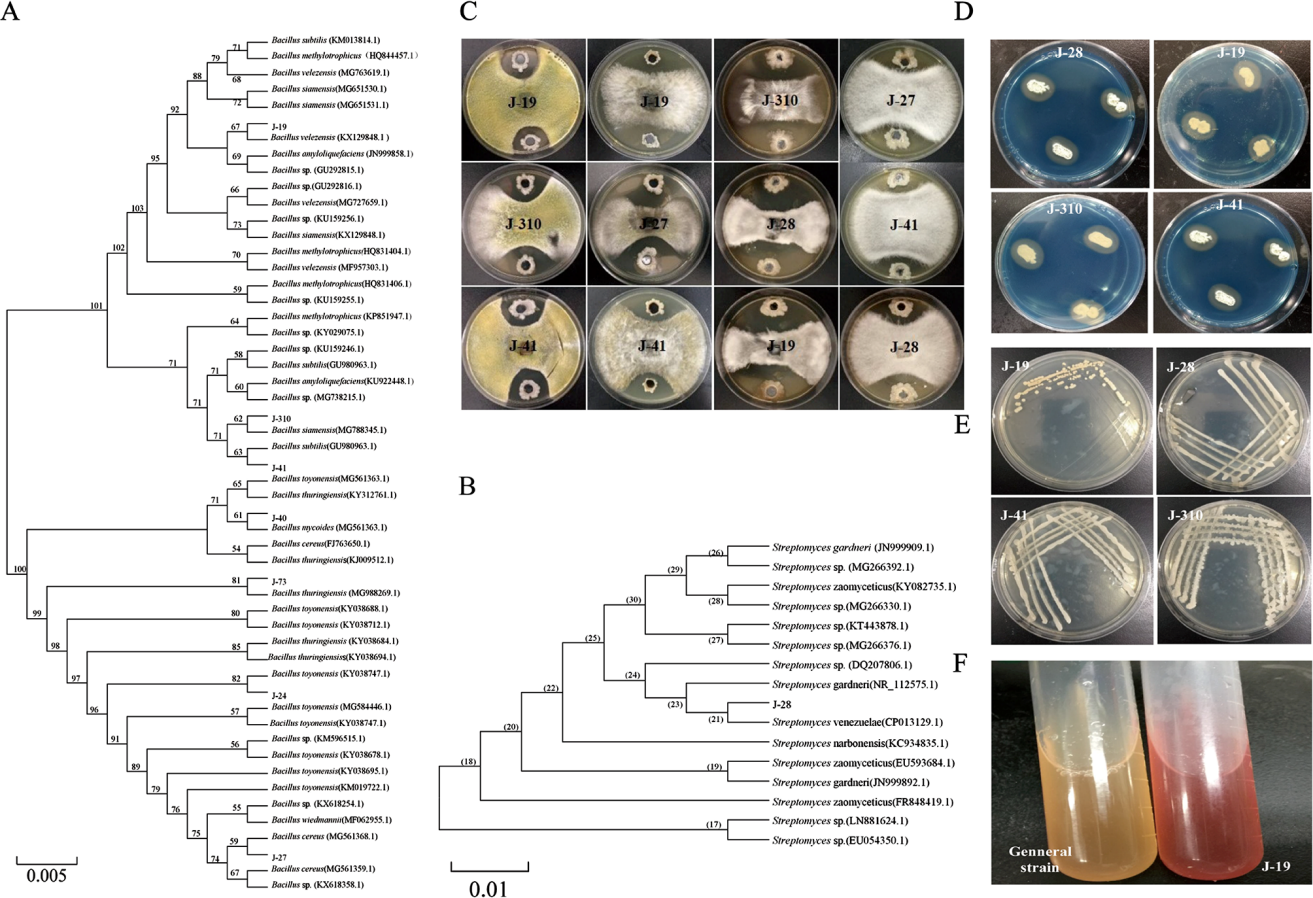
Identification and functional validation of eight antagonistic bacterial strains. (**A**) Phylogenetic tree of the *Bacillus* antagonistic strains based on 16 S rRNA gene sequence. (**A**) Phylogenetic tree of the *Streptomyces* antagonistic strains ased on 16 S rRNA gene sequence. (**C**) Plate confrontation experiment of the eight antagonistic strains, and from left to right, the fungi are: *Physalospora piricola*, *Rhizoctonia solani*, *Botryosphaeria ribis*, *Fusarium oxysporum*. (**D**) The color circle of different siderophore-producing strains in SA general plate. (**E**) Strains with ability producing ACC deaminase grow in ADF medium. (**F**) The Qualitative Detection of IAA (red: positive, yellow: negative)

The fungistatic effects of 353 bacterial strains isolated from the rhizosphere soil of apple trees in the Bohai Gulf were tested against *Fusarium oxysporum*, *Rhizoctonia solani*, *Botryosphaeria ribis*, and *Physalospora piricola* (Fig. 1). The double antibody labeling method was used to determine the colonization of the marked strains and the PGP ability of these antagonistic strains was verified. Based on the results, eight strains (J-73, J-19, J-310, J-24, J-27, J-28, J-40, J-41) with stable colonization ability, strong antagonistic effects against the four pathogens, and PGP were selected.

All eight strains showed remarkably high antifungal effects against the four pathogens (Table S1). However, the fungistatic effect among the different strains, determined based on zone of inhibition, differed, being 6.52–11.22 mm for *Fusarium oxysporum*, 6.58–12.46 mm for *Botryosphaeria ribis*, 8.12–14.28 mm for *Rhizoctonia solani*, and 6.07–18.09 mm for *Physalospora piricola*. The eight antagonistic strains colonized the rhizosphere soil, with viable bacterial counts of 10^6^ CFU/g after 84 days of inoculation (Figure S1). The strains J-19, J-310, J-28, and J-41 are capable of producing siderophores and ACC deaminase; and strain J-19 also has the ability to produce IAA, underscoring their potential to enhance plant resistance against biotic stress and pathogenic attacks. Thus, these bacterial strains not only resist the four common pathogens but also have a potential PGP effect, suggesting that they could be used as the strains for the prevention and control of continuous cropping disorder for further research (Fig. 1D-F).

The eight isolates were further classified and identified. The alignment results showed that for strains J-73, J-19, J-310, J-24, J-27, J-40, and J-41, the genus with the nearest genetic distance was *Bacillus*, whose gene sequences with a homology of up to 99% for *Bacillus thuringiensis* (MG988269.1), *Bacillus velezensis* (KX129848.1), *Bacillus siamensis* (MG788345.1), *Bacillus toyonensis* (KY038747.1), *Bacillus cereus* (MG561368.1), *Bacillus mycoides* (MG561363.1), and *Bacillus subtilis* (GU980963.1). J-28 had 100% sequence identity with *Streptomyces venezuelae* (CP013129.1). Thus, J-73, J-19, J-310, J-24, J-27, J-40, J-41 are members of *Bacillus*, consistent with the high prevalence of Bacillaceae in the rhizosphere soil of apple and other Rosaceae, where they act as antagonists [44, 45]. J-28 belongs to *Streptomyces* (Fig. 1A and B).

There were no antagonistic interactions observed between these 8 highly antagonistic strains (Table S2). Synthetic communities that combine the functions of several strains of microbes are generally more stable than a single strain [46]. Thus, in this study, the eight strains with the ability to promote plant growth and inhibit pathogenic fungi were mixed in equal proportions to establish the SynCom.

### Evaluation of functional assemblages of microbial consortia

Next, the physicochemical properties of soils in which *Malus hupehensis* Rehd were grown under different treatments were analyzed (Table 1). In treatment A, AN was 123.73 mg/kg, AK was 155.08 mg/kg, AP was 57.01 mg/kg, and OM was 3.07%, which were higher than other treatments. And the SM (14.24%), AK (151.14 mg/kg), AP (42.80) mg/kg, and OM (2.40%) were the lowest in treatment P. Among them, AP and OM contents in treatment A were significantly higher than that in treatment P (*P* < 0.05).

**Table 1:** Soil physiochemical properties in the different treatments

Treatment	pH	SM (%)	OM (%)	AN (mg/kg)	AK (mg/kg)	AP (mg/kg)
S	6.89 ± 0.01c	19.20 ± 0.15c	2.81 ± 0.18ab	123.50 ± 6.54a	152.06 ± 2.43a	52.51 ± 3.81ab
P	6.34 ± 0.01d	16.24 ± 0.18d	2.40 ± 0.26b	117.43 ± 10.41a	151.14 ± 2.27a	42.80 ± 0.60b
M	6.94 ± 0.03b	21.37 ± 0.65b	2.99 ± 0.31a	115.75 ± 0.53a	154.58 ± 11.26a	46.70 ± 0.83ab
A	7.04 ± 0.01a	23.32 ± 0.43a	3.07 ± 0.06a	123.73 ± 4.85a	155.08 ± 1.18a	57.01 ± 9.74a

S (inactivated SynCom), P (pathogen only), M (pathogens and SynCom); A (SynCom only). Values represent means ± standard deviation (SD) of three replicates. Different letters (a, b, c) indicate statistically significant differences at *P* < 0.05. Abbreviations: pH (potential of hydrogen), SM (soil moisture), OM (organic matter), AN (available nitrogen), AK (available potassium), AP (available phosphorus).

Measurements of the height, root length, and number of leaves of *Malus hupehensis* Rehd potted seedlings showed significant differences among the four treatments (*P* < 0.05) (Table 2). The average plant height (cm) in treatments S, P, M, and A were 44.49, 29.91, 36.55, and 46.82; the average number of leaves was 21.33, 1.33, 18, and 27.33; and the average root length (cm) was 16.51, 13.14, 14.58, and 19.54, respectively. The plant height, root length and number of leaves in treatment A were higher than the other three treatments, whereas the root length and number of leaves in treatment M were significantly higher than that in treatment P (*P* < 0.05), suggesting a possible growth-promoting effect of SynCom on seedlings.

**Table 2:** Growth of the pot seedlings in different treatments

Treatment	Plant height (cm)	Root length (cm)	Number of leaves
S	44.49 ± 1.84b	16.51 ± 0.75a	21.33 ± 2.52b
P	29.91 ± 4.06c	13.14 ± 1.80c	1.33 ± 2.31c
M	36.56 ± 2.88bc	14.58 ± 1.30b	18.00 ± 1.00b
A	46.82 ± 0.88a	19.54 ± 1.39a	27.33 ± 1.53a

S (inactivated SynCom), P (pathogen only), M (pathogens and SynCom); A (SynCom only). Values represent means ± standard deviation (SD) of three replicates. Different letters (a, b, c) indicate statistically significant differences at *P* < 0.05.

### Microbial community structure and diversity under the different treatments

**Fig. 2:**
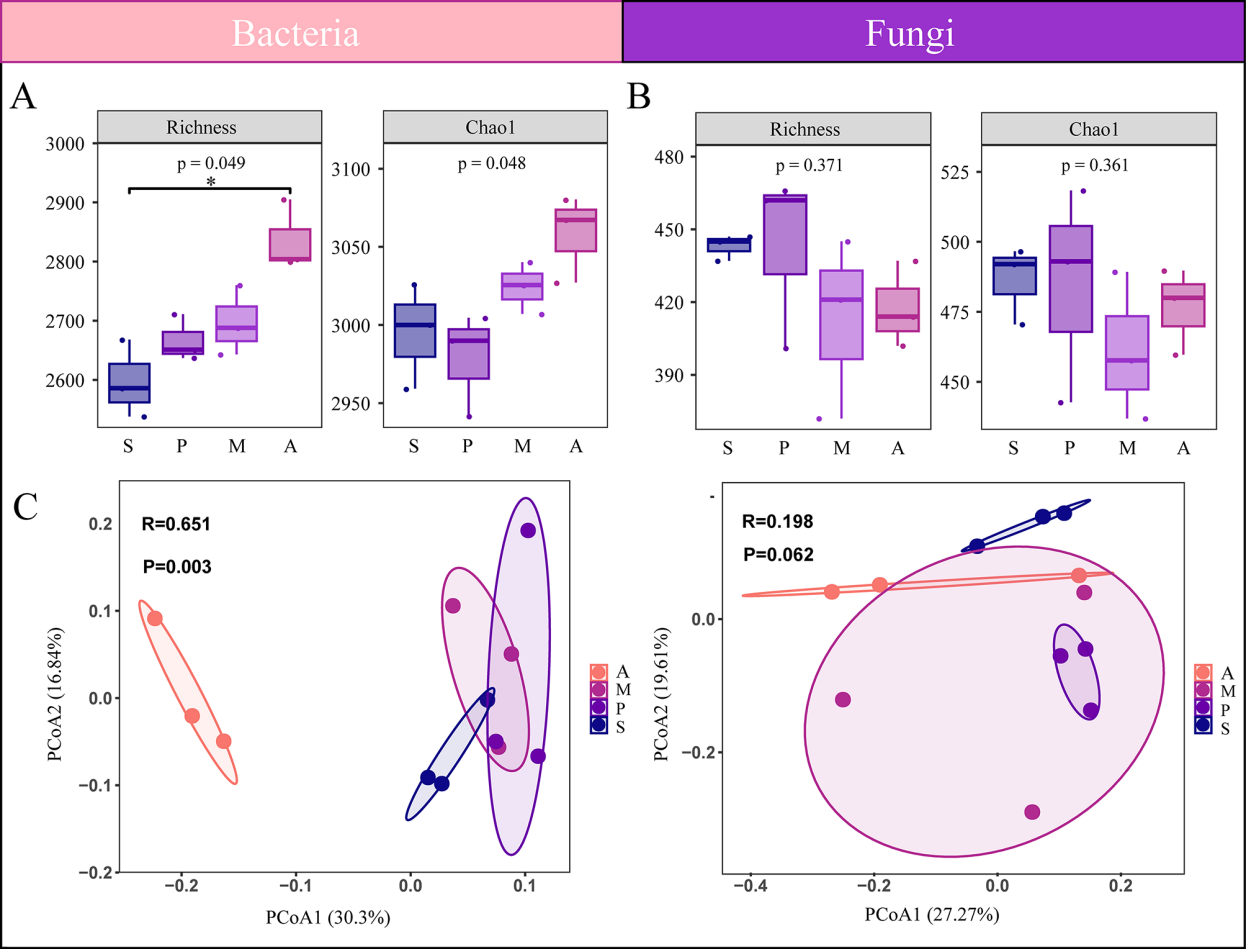
Microbial community structure and diversity. (**A**, **B**) Bacterial and fungal community alpha diversity indices in different treatments determined by Richness and Chao1 index. (**C**, **D**) PCoA (Principal Co-ordinates Analysis) represents microbial community structure of bacteria and fungi

According to the 16 S rRNA amplicon sequencing results, 72,871, 50,527, 47,639, and 49,350 bacterial clean tags were obtained in treatments A, S, P, and M, respectively. The alpha-diversity index was used to characterize the diversity of the microbial communities (Fig. 2). After the application of the SynCom, there was a significant impact on the species richness of the bacterial community. Treatment A had the highest richness index and Chao1, followed by treatment M, while treatments S and P had lower richness index and Chao1 (*P* < 0.05) (Fig. 2A). The ITS regions of fungi were analyzed, with 28,336, 34,889, 44,159, and 40,490 fungal clean tags obtained in treatments A, S, P, and M, respectively. The richness index and Chao1 were highest for treatment P, followed by treatment S, M, A (Fig. 2B).

The PCoA based on Bray-Curtis using the high-throughput sequencing data clearly distinguished between the four treatments for both bacteria and fungi. The fungal and bacterial compositions of the rhizosphere microbial communities clustered into distinct groups that well corresponded to the different treatments. The rhizospheric bacterial communities of *Malus hupehensis* Rehd exhibited significant differences among the four treatments (*P* < 0.05) (Fig. 2C, D). Treatment A exhibited noticeable differences in rhizospheric microbial composition with the other treatments, indicating that the addition of SynCom may cause the change and disturbance of plant rhizospheric soil bacterial community composition. In addition, the difference in rhizosphere bacterial community composition was greater than that in fungal community (*R* = 0.651 and *P* = 0.003 for bacteria, *R* = 0.198 and *P* = 0.062 for fungi). These findings demonstrate that SynCom had a greater effect on bacterial community than on fungi community, and that SynCom increased the richness of bacterial community.

### Dominant and differential genera under the different treatments

**Fig. 3:**
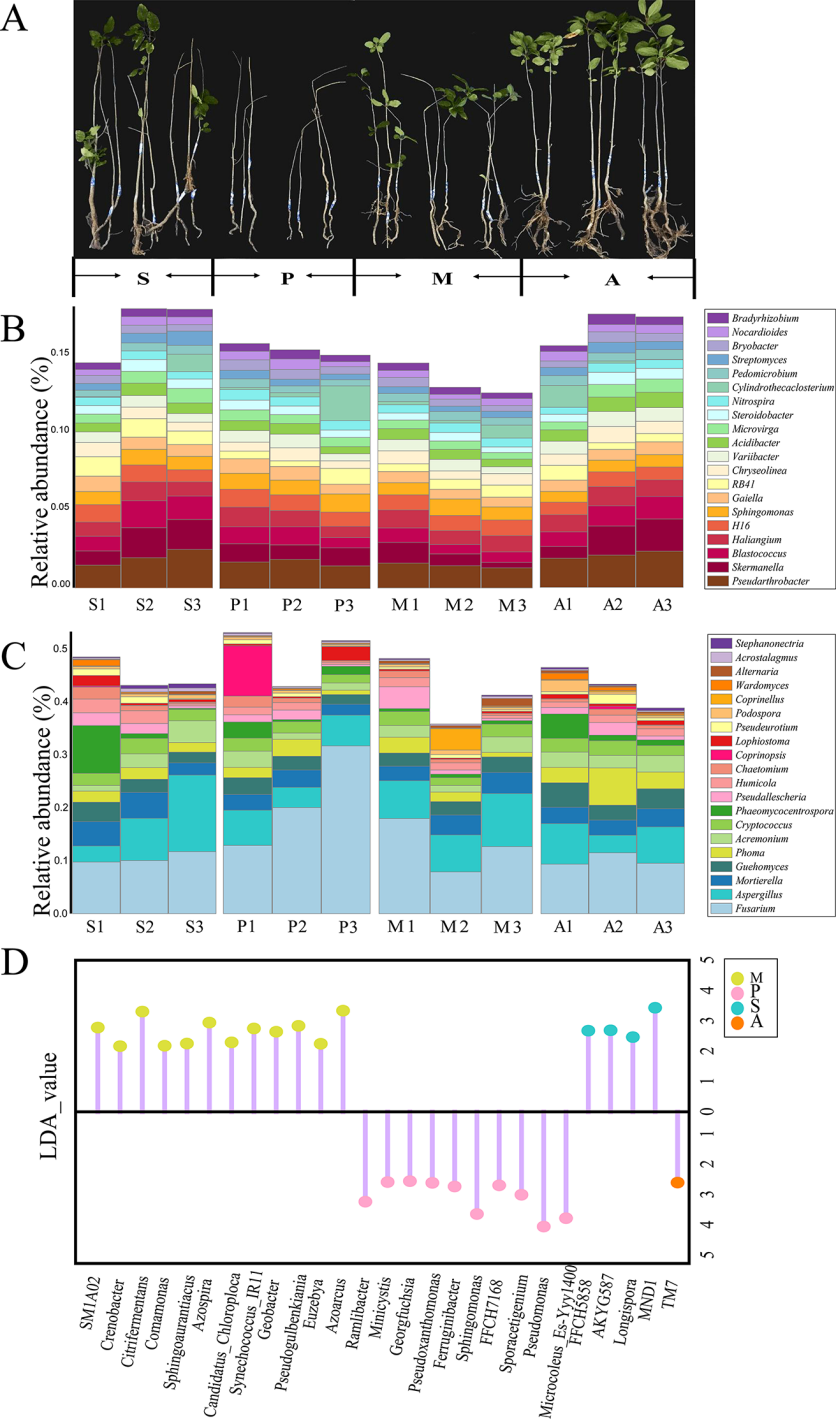
Seedling growth and microbial species under different treatments. (**A**) Seedling growth of four treatments at harvest. (**B**) Stacked bar chart of the relative abundance of the top 20 dominant bacterial genera. (**C**) Stacked bar chart of the relative abundance of the top 20 dominant fungal genera. (**D**) LDA Effect Size analysis (LEfSe) obtained bacterial biomarkers of different treatments

Changes in bacterial and fungal communities in response to exogenous microorganisms were evaluated by comparing the genus-level diversity of *Malus hupehensis* Rehd rhizosphere soil in the four treatments. The microbial diversity fluctuated during the different treatments (Fig. 3).

The dominant bacterial genera in all four treatments were *Pseudarthrobacter*, *Skermanella*, *Blastococcus*, *Haliangium*, *Sphingomonas*, and *Chryseolinea*. The relative abundances of *Pseudarthrobacter* (2.05%), *Haliangium* (1.12%), and *Chryseolinea* (0.84%) in treatment A were higher than in the other treatments (Fig. 3B). *Pseudarthrobacter* efficiently degrades crude oil and multi-benzene compounds [47]. Members of the genus *Chryseolinea* form a key group able to suppress disease-causing *Fusarium* [48]. While *Streptomyces* and *Bacillus* do not fall within the top 20 dominant genera, we also evaluated their enrichment across different treatments. We observed that the relative abundance of *Bacillus* was highest in treatment A (0.312%), followed by treatment M (0.305%) and treatment P (0.187%). Similarly, the relative abundance of *Streptomyces* in treatment A (0.571%) > M (0.520%) > P (0.455%). These results suggest an increase in the relative abundance of potentially beneficial bacteria in treatment A that improved the soil environment, promoting *Malus hupehensis* Rehd growth and controlling disease.

*Fusarium*, *Aspergillus*, *Mortierella*, *Phoma*, and *Acremonium* were the dominant fungal genera. The relative abundance of the pathogenic fungus *Fusarium*, which causes multiple soil-borne diseases and reduces crop yields, was highest in treatment P (23.3%). It indicates that pathogen application led to a high relative abundance of *Fusarium* in the soil. The relative abundances of *Phoma* and *Phaeomycocentrospora* in treatment M (2.02% and 0.71%) were lower than in the other treatments. *Phoma* are well-known plant disease agents but they are also pathogens of animals and humans [49]. Species of *Pseudocercospora* include plant pathogens, endophytes, and saprobes, and some have been used as biological control agents of weeds [50] (Fig. 3C).

To further investigate the effects of the different treatments on the composition of the microbial communities, the species composition under the different treatments was compared in a LEfSe analysis to identify species whose abundances significantly differed between the four groups (Fig. 3D). *SM1A02, Crenobacter, Citrifermentans, Comamonas, Sphingoaurantiacus, Azospira, Candidatus_Chloroploca, Synechococcus_IR11, Geobacter, Pseudogulbenkiania, Euzebya, Azoarcus* were among the biomarkers identified in treatment M. Some research indicates that *Comamonas, Sphingoaurantiacus*, and *Geobacter* are renowned for their ability to degrade complex organic compounds and pollutants, making them valuable for bioremediation processes [51]. Additionally, *Azospira, Pseudogulbenkiania*, and *Azoarcus* play a vital role in the soil nitrogen cycle, enhancing soil fertility and supporting plant growth [52]. The LEfSe analysis also identified *TM7* (tentatively named Saccharibacteria) as a biomarker in treatment A, and detected high abundances of *Ramlibacter, Minicystis, Georgfuchsia, Pseudoxanthomonas, Ferruginibacter, Sphingomonas, FFCH7168, Sporacetigenium, Pseudomonas, Microcoleus Es-Yyy1400* in treatment P.

The relative abundances of the dominant genera and the results of the LEfSe analysis together showed that pathogen application increased the presence of potentially pathogenic microorganisms in the rhizosphere soil, which may exacerbate the occurrence of plant diseases. The application of SynCom, however, enriched the beneficial bacteria in the rhizosphere soil, which may help plants resist the invasion of pathogenic microorganisms, in addition to enhancing soil disease resistance and improving the soil environment.

### Co-occurrence network analysis and the evaluation of the microbial assembly process under the different treatments

**Fig. 4:**
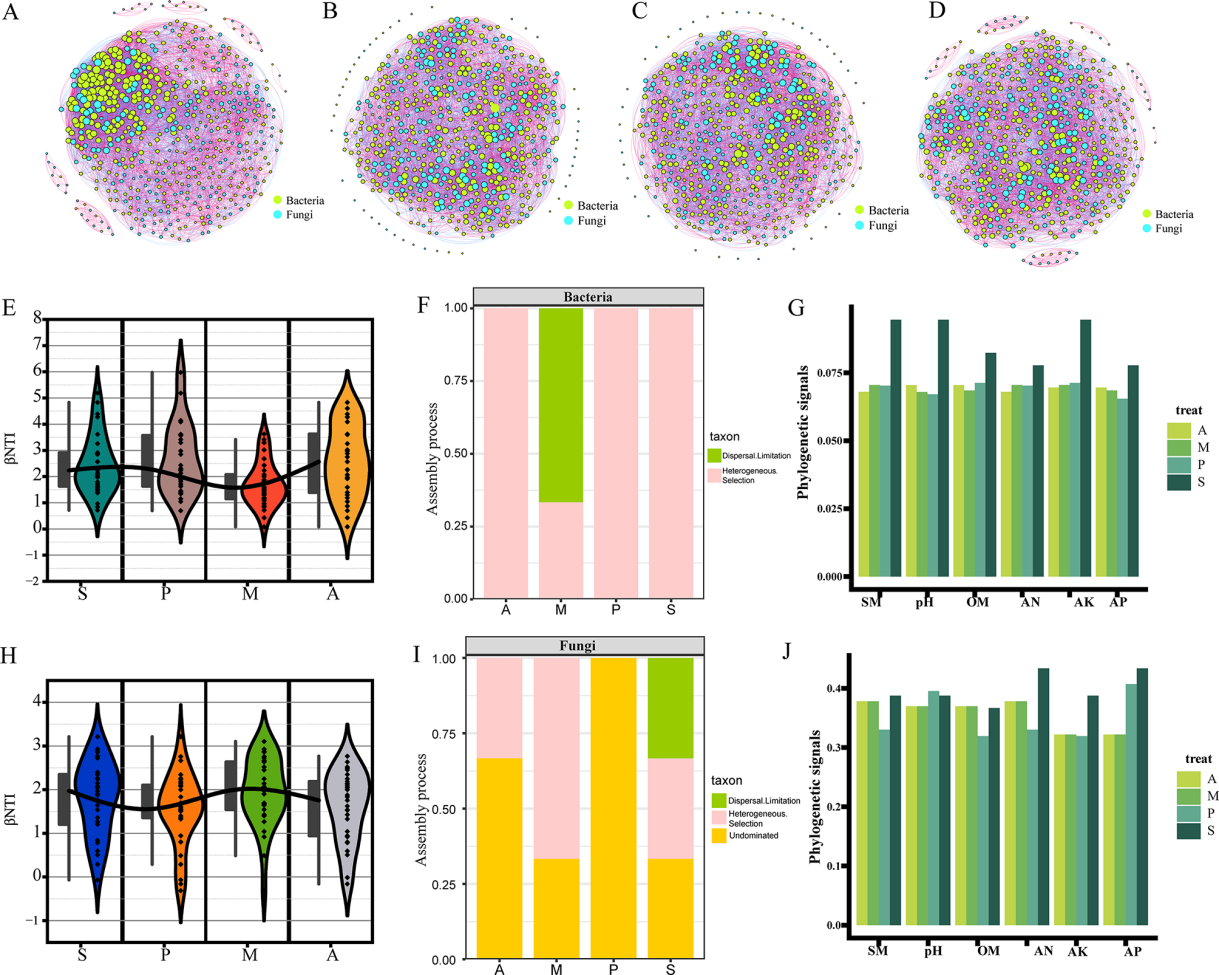
Co-occurrence networks and community assembly of bacteria and fungi. (**A**, **B**, **C**, **D**) Co-occurrence patterns of bacterial-fungal interkingdom networks in the rhizosphere soil of treatment S, P, M and A. The two colors represent bacteria and fungi, the red line represents positive correlation, the blue line represents negative correlation, and the size of the dot represents the height of the degree. (**E**, **F**) Formation (assembly) mechanism of the four treated bacterial communities. (**G**) Phylogenetic signals show the conservation of the environmental preference for bacterial communities by using Blomberg’s K value. (**H**, **I**) Formation (assembly) mechanism of the four treated fungal communities. (**J**) Phylogenetic signals show the conservation of the environmental preference for fungal communities by using Blomberg’s K value

The concept of an “integrative microbiome,” which includes protists, fungi, bacteria, archaea, and viruses, has been proposed as a direction for future microbiome studies [53]. Thus, in this study, linkages between bacterial and fungal communities and their interactions were investigated by constructing bacterial-fungal interkingdom networks. A metacommunity co-occurrence network of the relationships among bacteria and fungi based on Spearman’s correlation coefficients was established to examine the effects of exogenous strains on the rhizosphere microbial community and the co-occurrence patterns in treatments S, A, M, and P (Fig. 4). The number of edges, the average degree, the graph density, and the average clustering coefficient were highest in treatment A (SynCom application), suggesting a strongly correlated and complex network with the most links. The modularity of the co-occurrence network was the highest in treatment M (11.916), indicative of the “small-world” properties and nonrandom topology of its network. The bacterial-fungal interkingdom networks in treatments A and M had the same proportions of positive (64%) and negative edges (36%), while the co-occurrence networks in treatment P had remarkably large negative edge proportions (> 47%), which implies an increase in mutual exclusion rather than the coexistence of bacteria and fungi during pathogen treatment. These results further suggest that pathogen application to the rhizosphere soil stimulated strong negative interactions within the microbial community (Fig. 4A, B, C and D).

The influence of the exogenous SynCom on microbial community assembly was further explored by comparing the relative importance of determinism and stochasticity. Bacterial community assembly processes were quantified using the null model, which indicated the dominance of deterministic processes. The weighted microbial community assembly (βNTI) metric provides insights into the potential roles of deterministic and stochastic forces in the phylogenetic community dynamics of microbial communities. Significant deviations of |β NTI| > 2 are defined as indicating the dominance of deterministic processes, and deviations |β NTI| < 2 are considered to indicate the dominance of stochastic processes [54]. The community assembly of bacteria in treatments S, P, and A was mainly shaped by deterministic processes whereas the turnover of bacteria in treatment M was determined by both deterministic (33.33%) and stochastic (66.67%) processes (Fig. 4E and F). The assembly of fungal communities in treatment P was dominated by nondominant processes and was stochastic, whereas in treatments M and A the fungal communities were mainly influenced by heterogeneous selection and nondominant processes. In treatment S, the fungal community was determined by heterogeneous selection (33.33%), dispersal limitation (33.33%), and undominated processes (33.33%) (Fig. 4H and I).

A comparison of phylogenetic signals resulting from the four treatments further demonstrated the differential responses of aggregate communities to physicochemical variables (Fig. 4G and J). The bacterial community in control treatment S had the greatest phylogenetic signal for SM, pH, OM, AN, AK, and AP, indicating that it was more conservative in its phylogeny and less susceptible to external factors, while the addition of exogenous microbes led to a perturbation of the soil habitat and a reduction in environmental preferences. Compared to bacteria, the fungal community had a higher Blomberg’s K value and a more conservative phylogeny, indicative of a wider range of environmental adaptations. The phylogenetic signals resulting from treatments A and M, both of which included SynCom, were similar and suggested that the SynCom-treated soils contained microbial species with similar ecological preferences for specific physicochemical variables.

In summary, treatments A and M had similar bacterial-fungal interkingdom networks and phylogenetic signals. Their microbial networks appeared more stable, whereas treatment P displayed a higher level of negative interactions, such as competition and antagonism.

### Influence of SynCom on the soil microbial community of Malus hupehensis Rehd

**Fig. 5:**
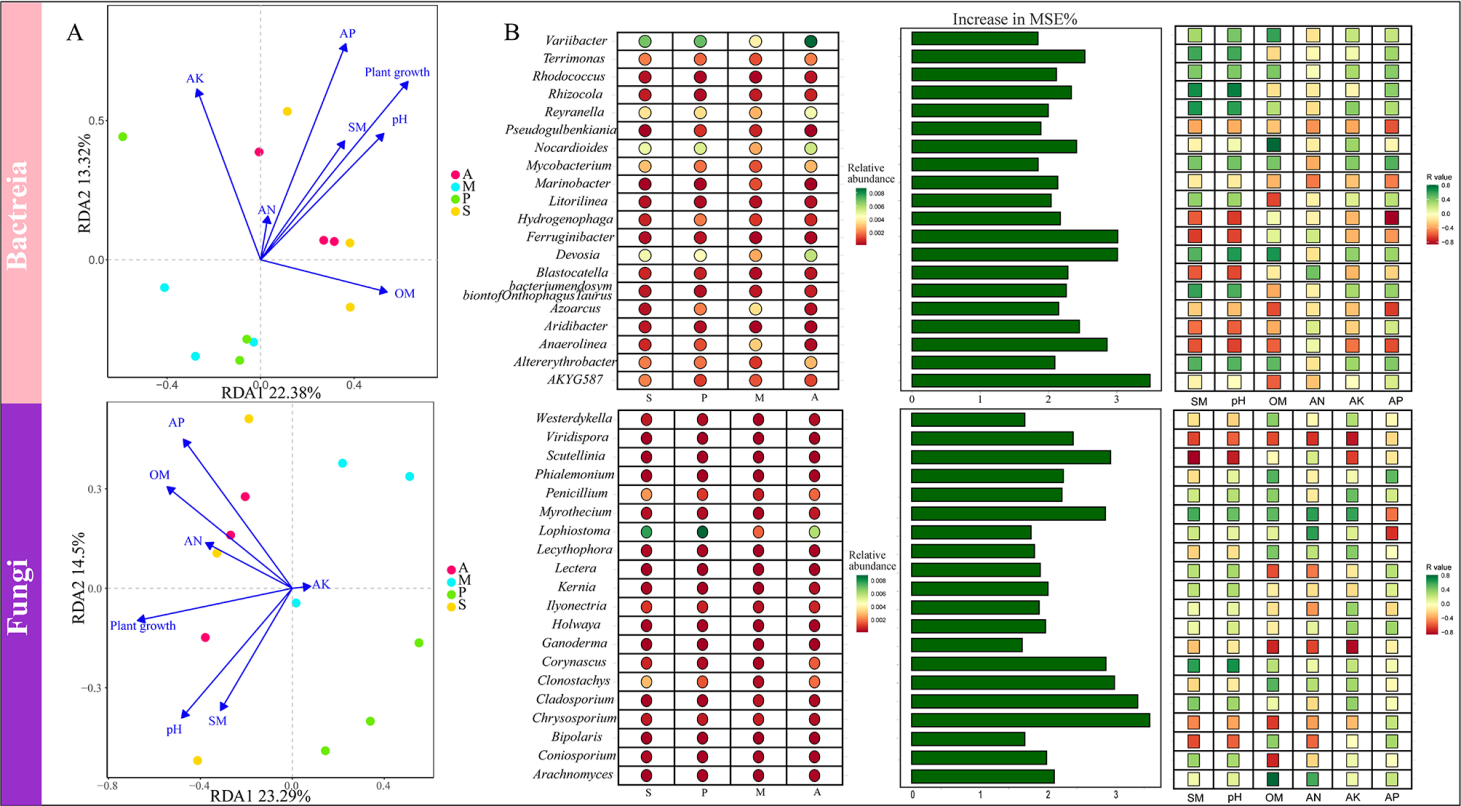
Environmental factors drive microbial communities and biomarkers. (**A**) RDA was used to analyze the correlation between environmental factors and microorganisms. (**B**) Random forest was used to find biomarkers and environmental drivers. The bubble chart shows the abundance of the Biomarker among the four groups of samples and the bar chart shows the importance of the top 20 genus, and the correlation matrix shows the association between the top 20 genus and environmental factors

The relationship between microbial communities and environmental factors under the different treatments was evaluated in a redundancy analysis (RDA) of the correlation between the bacterial and fungal communities and environmental factors in each treatment (Fig. 5).

The results indicated differences between treatments following application of the microorganisms. This was particularly evident in treatment A, treated only with the SynCom, which showed a positive correlation with environmental factors, particularly AP, pH, OM, and plant growth, and in the control treatment S, for which a positive correlation with environmental factors was also determined. Treatment A with antagonistic bacteria showed similar correlations with environmental factors to treatment S, indicating similar responses of their microbial communities to environmental factors. By contrast, treatments P and M, which included the pathogens, correlated negatively with environmental factors and plant growth except AK. The similar correlations of treatments P and M with environmental factors indicated similar responses of the microbial communities to environmental factors (Fig. 5A).

A model of the relationship between microbial community composition and each environmental factor was developed using the random forest algorithm to regress the relative abundance of each microbial genus against the environmental factors. Based on the importance values of the microbial genera, the top 20 bacterial genera and fungal genera were obtained. Among the former, most showed positive correlations with AP, pH, and OM (Fig. 5B). Among them, *Reyranella*, *Variibacter*, *Nocardioides*, *Devosia*, and *Altererythrobacter* had higher relative abundances in treatment A than in the other treatments. *Nocardioides*, which is arsenic- and antimony-resistant and thus of interest in the remediation of heavy-metal-contaminated sites [55], and *Devosia* may be effective for controlling plant diseases such as *Fusarium* head blight [56]. However, *Hydrogenophaga*, *Anaerolinea*, and *Azoarcus* correlated significantly and negatively with AP (*P* < 0.05). *Anaerolinea* and *Azoarcus* had high relative abundances in treatment M, with *Azoarcus* identified as a biomarker of this treatment in the LEfSe analysis. Both *Anaerolinea* and *Azoarcus* drive soil C, N, P, and S cycles in forests, improve arsenic contamination of soil, and remediate the soil environment [57, 58]. Among the 20 fungal genera, *Lophiostoma* had a high relative abundance in treatment P and also correlated negatively with AP (Fig. 5B). This genus is responsible for the formation of *Lophiostoma carpini* in woody plants [59].

### Prediction of bacterial and fungal functional profiles in rhizosphere soil after inoculation

**Fig. 6:**
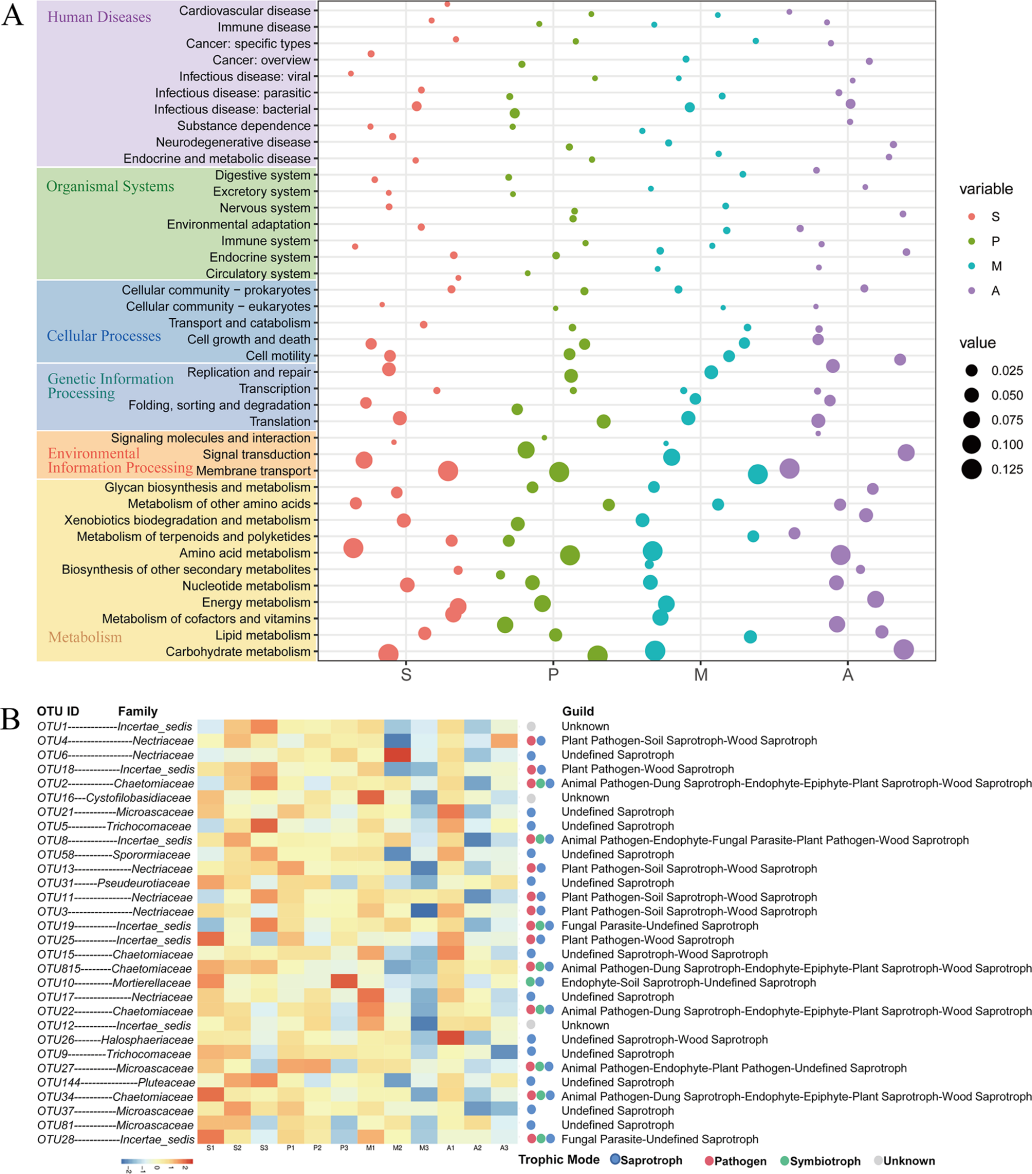
Variations in bacterial and fungal functional profiles in four treatments were analysed using PICRUSt and FUNGuild. (**A**) KEGG pathway annotation based on the KEGG database [36, 41, 42]. (**B**) Guild of fungal species with top 30 OTUs annotated by FUNGuild

To investigate whether the application of SynCom changed the functions of the bacterial and fungal communities, bacterial community function was predicted using the PICRUSt software and fungal community function was predicted using the FUNGuild tool of Majorbio Cloud. The functional spectra of the bacterial and fungal communities in the rhizosphere differed among the four treatments (Fig. 6).

The potential functions of the bacterial community were further examined by annotating the sequences according to KEGG pathways, which predicted metabolism (11 pathways), disease (10 pathways), biological systems (7 pathways), cellular processes (5 pathways), genetic information processing (4 pathways), and environmental information processing (3 pathways). The relative abundances of the energy metabolism, amino acid metabolism, transport, and catabolism, substance dependence, and cell growth pathways were significantly higher in treatment A than in the other treatments (*P* < 0.05). The relative abundance of the infectious disease and immune system pathway was highest in treatment P, which suggests that pathogen application may stimulated the pathogenic pathways of the fungal community, leading to the development of soil-borne diseases that in turn stimulated the functional genes of the immune system of the bacterial community, enhancing soil antagonism (Fig. 6A).

The results of FUNGuild analysis showed that 620 OTUs could be annotated with different trophic modes, accounting for 52.36% of the total OTUs, with some OTUs annotated with multiple trophic modes (Fig. 6B). Saprotrophs were represented by 531 OTUs, accounting for 44.85% of the total OTUs; this group is the dominant fungal trophic mode in the composting process. OTUs representative of symbiotrophs and pathotrophs accounted for 15.63% and 18.33%, respectively. For 60% of the OTUs, annotation to the family level was possible. The 30 OTUs with an average abundance greater than 0.1% were screened for their predictive functional information and abundances using a heatmap. Most of the OTUs associated with pathogenic pathways tended to show high relative abundance in treatment P, such as OTU27, OTU6, and OTU9. OTU27, which belongs to Microascaceae, which are plant pathogens such as *Microascus cirrosus* that may affect plant health and crop yield [60]. OTU6 belongs to Nectriaceae, a family containing important plant pathogens. OTU9 was assigned to saprotrophic fungi including *Aspergillus_subversicolor* and the *Aspergillus* genus. Some versicolores species are facultative human and animal pathogens [61]. Their abundances in treatments A and M were low, indicating a decrease in the relative abundance of harmful fungi in the rhizosphere after SynCom application.

## Discussion

Plants have evolved to attract microbes that promote plant growth and development from soils to their roots [62]. Plant-associated microbiota can influence the disease resistance, nutrient status, growth rate, and stress tolerance of their host plants [63]. In this study, eight strains were isolated that showed antagonistic ability against *Fusarium oxysporum*, *Rhizoctonia solani*, *Botryosphaeria ribis*, and *Physalospora piricola*, able to stably colonize, and some of them can produce siderophores, ACC deaminase, and IAA. Siderophore and IAA production are associated with growth promotion in host plants while ACC deaminase reduces ethylene levels, thereby enhancing plant growth [64, 65].

**Fig. 7:**
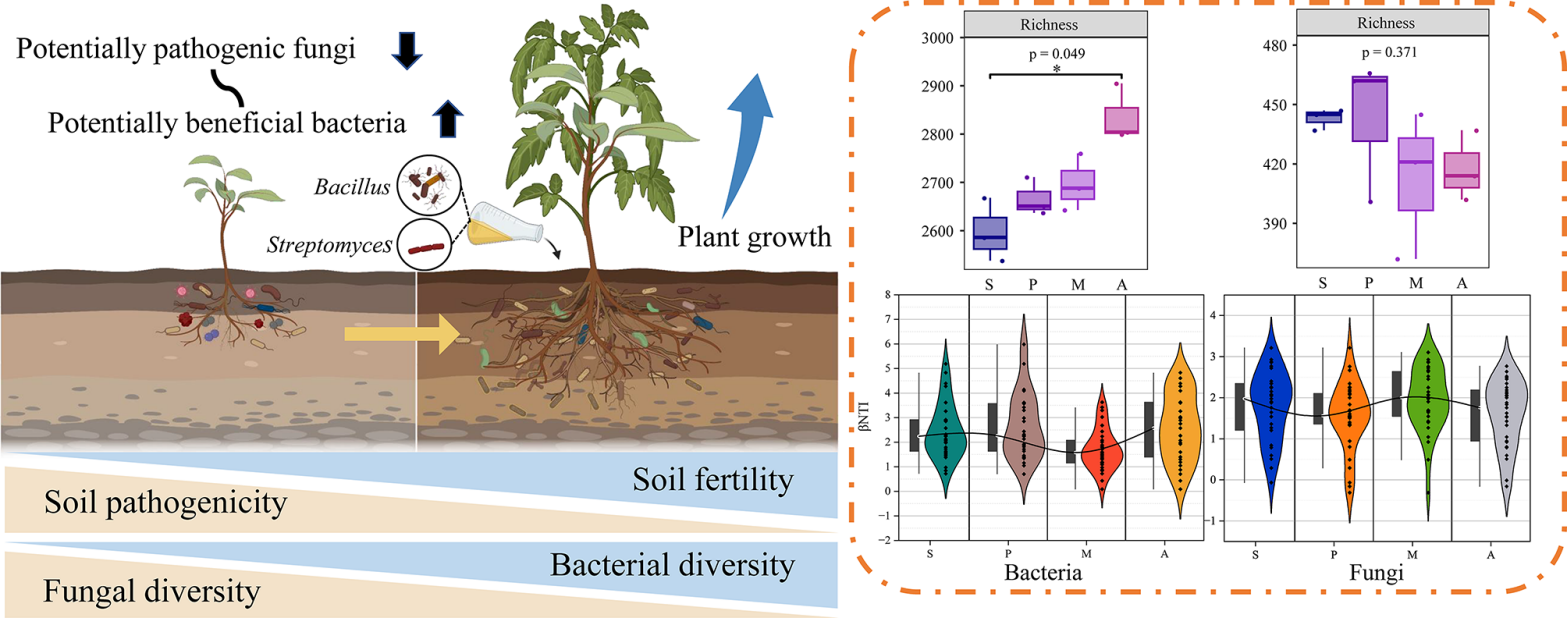
Effects of SynCom on the growth of *Malus hupehensis* Rehd and its microbial community

These eight beneficial microorganisms were used to construct a SynCom, and its effects and mechanisms of action were investigated (Fig. 7). The eight strains belonged to the genera *Bacillus* and *Streptomyces*, which are among the root-associated bacterial genera identified in previous studies, together with *Azospirillum* and *Pseudomonas*. Isolates from these have already been deployed as biofertilizers [66]. Both Bacillaceae and Streptomycetaceae are considered plant growth promoting rhizobacteria (PGPR), and they are specifically recruited by plants to suppress disease [16]. Their effects on disease control and host plant performance were the basis for constructing a SynCom that was used in pot experiments. The SynCom led to an increase in the soil pH. Previous have shown that increased soil acidity inhibits the activity of some soil microorganisms, thereby affecting the conversion and utilization of nitrogen and other nutrients [67]. The SynCom also increased the levels of available nutrients in the soil, including AP and organic matter, most likely by recruiting beneficial microorganisms such as phosphate-solubilizing, potassium-solubilizing, and nitrogen-fixing bacteria. Such microbes can convert insoluble minerals and organic matter into a continuous supply of available nutrients. These microorganisms may also have induced the plants to secrete root exudates that in combination with colloids formed a soil aggregate structure that improved the porosity, water and fertilizer retention, and general properties of soils. This would provide a suitable environment for microbial metabolism and soil enzymes, thereby enhancing the circulation and utilization of nutrients [68]. Soil microbes contribute to nutrient enrichment and play a crucial role in the modulation of primary production by controlling the decomposition and availability of nutrients as well as root grazing and plant nutrient absorption to maintain soil productivity [69]. SynCom promoted the growth of *Malus hupehensis* Rehd, including rhizome elongation and an increase in the number of leaves, all of which provide plants with advantages in terms of nutrient competition, resource access, and vitality. Plants attract growth-promoting microorganisms to their root system by releasing exudates like sugars, organic acids, amino acids, and phenolics. These microorganisms play a role in enhancing plant growth through nutrient transformation, translocation, and the regulation of phytohormones such as auxins, cytokinins, gibberellins, abscisic acid, and ethylene [70].

The composition and structure of the rhizosphere soil microbial community were also affected by the application of SynCom. Soil biodiversity in general, and microbial diversity in particular, is a driving force underlying the soil processes that are essential to sustain agricultural production [71]. The results showed an increase in the richness of bacteria after the application of SynCom. The increase of bacteria and the decrease of fungi in soil after the application of SynCom is an important direction for the control of successive cropping obstacle [72]. The rhizosphere bacterial community structure of treatment A was significantly separated from that of other treatments, indicating that SynCom application may cause the change of the bacterial community. Studies have shown that soil bacterial communities are more sensitive than fungi and are easily affected by external factors [73, 74]. In treatment A and M, the relative abundances of potentially beneficial microorganisms in the dominant genera, such as *Pseudarthrobacter, Haliangium*, *Chryseolinea*, *Streptomyces* and *Bacillus*, were increased. *Pseudarthrobacter* efficiently degrades crude oil and multi-benzene compounds. *Haliangium* species are the core bacteria in the rhizosphere of *ex situ* wild rice and play an important role in improving nutrient resource acquisition for rice growth [75]. These microbes degrade crude oil and multi-benzene compounds and suppress disease-causing *Fusarium*, respectively [48, 76]. And the *Bacillus* and *Streptomyces* are well-recognized Plant Growth-Promoting Rhizobacteria (PGPRs) with well-documented beneficial effects. In treatment M, the relative abundances of the potentially pathogenic fungi *Phoma* and *Phaeomycocentrospora* were reduced while those of bacteria involved in disease suppression and environmental remediation, such as *Azoarcus*, *Geobacter*, *Azospira*, *Pseudogulbenkiania*, and *SM1A02* were increased. Both the LEfSe analysis and the random forest model predictions identified *Azoarcus* as a biomarker for treatment M. This genus, along with *Gluconacetobacter* and *Herbaspirillum*, is among the nitrogen-fixing bacteria found in disease-free host plant tissues [77]. *Azoarcus*, *Geobacter*, and *Azospira* are nitrogen-fixing, converting nitrogen in the air into ammonium salt available for plants, thus providing an essential nutrient for plant growth and development, significantly improving the nitrogen supply capacity of soil, and reducing soil electrical conductivity. Together, these activities improve the microecological environment of plant roots [78]. The abundance of the pathogenic fungus *Fusarium*, which causes multiple soil-borne diseases and reduces crop yields, was highest in treatment P [79]. Most *Fusarium* species are plant pathogens, such as *F*. *maniliforme*, *F*. *solani* and *F*. *xysporum*; their abundance in the pathogen-treated rhizosphere soil was as high as 16% [80, 81]. Taken together, our results suggest that the SynCom developed in this study enhances the soil environment by suppressing soil-borne, disease-causing fungi and recruiting antagonistic bacteria and growth-promoting microorganisms. This collective action contributes to controlling pathogens associated with apple disease and supports the maintenance of healthy plant growth. Network topological parameters can be used to characterize the complexity and stability of the network [82]. According to the co-occurrence patterns in the bacterial-fungal interkingdom networks, the number of edges, average degree, graph density, and average clustering coefficient of treatment A indicated that SynCom application stabilized the microbial community. The highest modularity coefficient (11.916) was found in treatment M, such that the antagonistic effect of SynCom on pathogens may have strengthened the clustering intensity of the bacterial and fungal communities, with a clearer association structure. The SynCom induced interactions among microbial communities, enhancing the stability of the microbial network. Microbial community assembly processes were also investigated. Deterministic processes are those in which abiotic and biotic factors determine the presence or absence and relative abundance of a species and thus are related to ecological selection. Stochastic processes include random changes in the probability distribution and relative abundance of species (ecological drift) that are not the result of adaptations to the environment [83]. Tao et al. found that the assembly of healthy soil bacterial communities in soil was mainly a stochastic process [84]. In our study, the assembly of bacterial community groups in treatment M was a stochastic process, whereas in treatment P assembly was a deterministic process. These results imply that the SynCom was able to confer resistance to pathogen invasion and drive the transformation of diseased soil into healthy soil.

The physical and chemical properties of soil affect the structure and function of microorganisms and their interaction with plants, such that changes in soil structure can have a profound effect on the survival and metabolism of soil microorganisms. Conversely, microbes influence soil fertility and nutrient turnover through the secretion of metabolites and enzymes, which in turn can affect crop growth [85]. In accordance with previous results, the amount of available nutrients in the soil increased after the application of SynCom. Among environmental factors, AP and OM were the main limiting factors for microbial communities and both were positively correlated with treatments A and S. Phosphorus is usually the limiting nutrient in agroecosystems. Microbial biomass itself is a large and dynamic phosphorus reservoir that responds rapidly to environmental changes and can drive phosphorus effectiveness through transfer and fixation mechanisms; it is therefore an important regulator of phosphorus effectiveness [55]. The level of organic matter in soil impacts the structure of microbial communities, and carbon sources are a key ecological driver of microbial community dynamics [86]. The high-level growth achieved with treatment A shows that increasing organic matter and AP may improve field plantings of apple seedlings.

## Conclusions

Crop cultivation under disease stress can be improved utilizing microbiomes from this environment. In this study, a SynCom promoted plant growth and increased the nutrient content of the soil, including organic matter and AP. It also increased the diversity of bacteria as well as the relative abundances of potentially beneficial bacteria while decreasing the relative abundance of potentially pathogenic microorganisms, in the rhizosphere. It also improved the stability of the microbial community of the rhizosphere, promoting the growth of apple plants. Similar functional synthetic communities can be used to achieve sustainable agriculture, promoting plant growth while avoiding common apple diseases.

### Electronic supplementary material

Below is the link to the electronic supplementary material.

Supplementary Material 1

## Data Availability

Sequence data that support the findings of this study have been deposited in the Sequence Read Archive at NCBI with the accession number PRJNA1009678.
