# Supplementary material for: Plant growth promotion and biocontrol properties of a synthetic community in the control of apple disease
Source: BMC Plant Biol. 2024 Jun 13;24:546. doi: 10.1186/s12870-024-05253-8 (PMC11177370; doi:10.1186/s12870-024-05253-8)
Supplement: Supplementary file 1 — Supplementary Material 1 [file 12870_2024_5253_MOESM1_ESM.docx]

Supplementary Material

# Supplementary Table

**Table S1** Zone of inhibition (mm) of select bacterial isolates against 4 different fungal pathogens of apple

| Strain | *Fusarium oxysporum* | *Botryosphaeria*  *ribis* | *Rhizoctonia*  *solani* | *Physalospora piricola* |
| --- | --- | --- | --- | --- |
| J-19 | 9.54±0.16 | 10.62±0.74 | 14.28±0.54 | 18.09±1.28 |
| J-310 | 7.08±1.15 | 12.46±0.02 | 11.08±0.32 | 17.28±1.96 |
| J-73 | 6.52±0.16 | 11.15±0.69 | 8.12±0.58 | 13.76±2.36 |
| J-24 | 9.54±1.94 | 8.33±0.12 | 9.01±2.37 | 6.07±0.03 |
| J-27 | 11.22±1.96 | 6.58±0.44 | 14.23±0.99 | 7.40±1.94 |
| J-28 | 10.57±0.03 | 11.95±2.65 | 11.29±0.43 | 14.94±1.415 |
| J-40 | 7.24±1.28 | 8.01±2.23 | 10.87±0.97 | 10.00±2.40 |
| J-41 | 10.81±0.57 | 10.60±1.10 | 13.08±0.22 | 17.15±2.17 |

The zone of inhibition (ZOI) was precisely measured from the center of the bacterial colony to the leading edge of the fungal growth inhibition, showcasing the antibacterial activity of the strains.

**Table S2** Antagonistic results among the 8 antagonistic strains

| Strains | J-19 | J-24 | J-24 | J-28 | J-40 | J-41 | J-73 | J-310 |
| --- | --- | --- | --- | --- | --- | --- | --- | --- |
| J-19 | - | - | - | - | - | - | - | - |
| J-24 | - | - | - | - | - | - | - | - |
| J-24 | - | - | - | - | - | - | - | - |
| J-28 | - | - | - | - | - | - | - | - |
| J-40 | - | - | - | - | - | - | - | - |
| J-41 | - | - | - | - | - | - | - | - |
| J-73 | - | - | - | - | - | - | - | - |
| J-310 | - | - | - | - | - | - | - | - |

"-" indicates no antagonism to each other.

**Table S3** Topology parameters of co-occurring networks with different treatments

| Treatment | Positive connection | Negative connection | Node | Edge | Average degree | Average weighted degree | Graph density | Modularity | Average clustering coefficient |
| --- | --- | --- | --- | --- | --- | --- | --- | --- | --- |
| S | 61% | 39% | 573 | 10790 | 36.221 | 3.592 | 0.066 | 6.899 | 0.502 |
| P | 52% | 47% | 572 | 10088 | 34.967 | 1.479 | 0.061 | 8.537 | 0.461 |
| M | 58% | 42% | 573 | 10615 | 37.051 | 1.208 | 0.065 | 11.916 | 0.459 |
| A | 63% | 36% | 577 | 10970 | 38.024 | 4.997 | 0.066 | 4.899 | 0.511 |

# Supplementary Figure


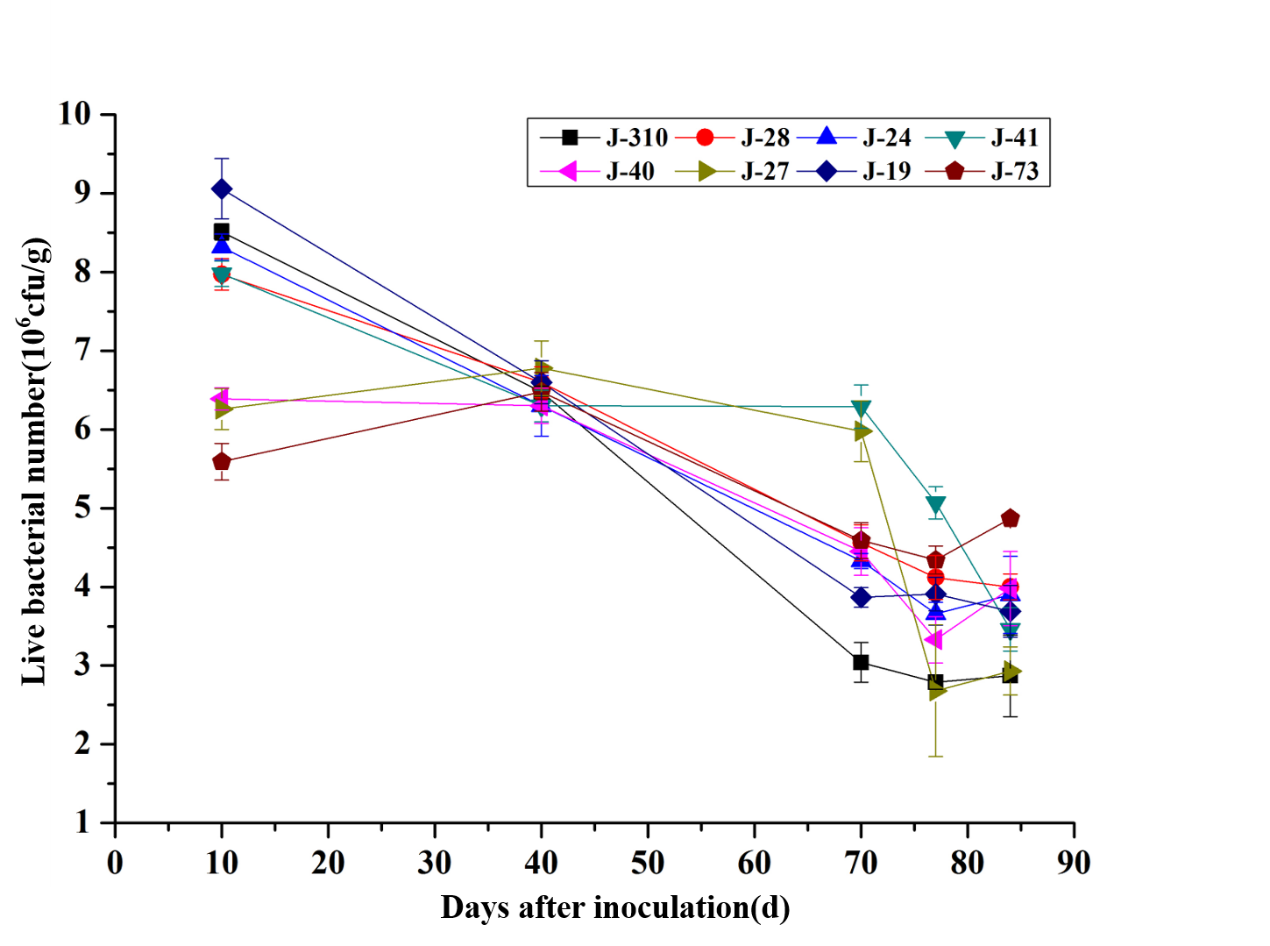


**Figure S1** Growth and decline trend of marked bacteria in the rhizosphere soil
